# Analytical Validation of NETest2.0^®^, a Novel Multigene Blood-Based Molecular Assay for Neuroendocrine Tumors

**DOI:** 10.3390/cancers18111719

**Published:** 2026-05-25

**Authors:** Mark Kidd, Srinivas V. Koduru, Abdel Halim

**Affiliations:** Wren Laboratories, Branford, CT 06405, USA; skoduru@wrenlaboratories.com

**Keywords:** blood, liquid biopsy, NETest2.0^®^, PCR, analytical validation, algorithm, cutoff, assay performance

## Abstract

**Background/Objectives**: Blood-based RNA diagnostics requires rigorous analytical validation prior to clinical implementation. NETest2.0^®^ is a machine learning-enhanced 55-gene mRNA assay designed to detect neuroendocrine tumor (NET)-associated gene expression signatures in peripheral blood. **Methods**: Analytical performance was evaluated in accordance with CLSI EP05, EP06, EP07, and EP17 guidelines and ISTA 7D 2007 standards. Accuracy was assessed in 973 samples (568 NETs, 219 non-NET cancers, 186 controls). Precision, sensitivity, linearity, and specificity were evaluated using cell line-spiked blood, patient samples, and interference testing. Stability and robustness were assessed under varying storage and transport conditions. **Results**: NETest2.0^®^ demonstrated > 90% diagnostic accuracy. All transcripts were consistently amplified (Ct < 35; mean efficiency 1.94). Intra-assay coefficients of variation (CVs) were 0.56% (Ct) and 2.07% (score), while inter-assay CVs were 4.12% and 6.85%, respectively. High concordance across operators and instruments was observed (r = 0.82–0.94, *p* < 0.0001). The limit of detection was 2.2 cells/mL RNA-equivalent, with 95% detection at 2.29 cells/mL; the limit of quantification was 8.6 cells/mL. Assay output was linear across defined ranges and unaffected by endogenous or exogenous interferents. No associations were identified with demographic, hematologic, renal, or hepatic variables. Samples remained stable at ambient temperature for up to 10 days and at −80 °C for up to 5 years, with no impact from shipping conditions. **Conclusions**: NETest2.0^®^ demonstrates high analytical sensitivity, precision, and robustness, supporting its validity for clinical application in NET management.

## Simple Summary

This study demonstrates that the novel blood-based multigene qPCR NETest2.0^®^ assay has a low limit of detection (approximately 2 tumor cells RNA-equivalent/mL), strong intra- and inter-assay reproducibility, and resistance to common endogenous and exogenous interferents, including medications and metabolic factors. Importantly, the assay performance was unaffected by age, sex, ethnicity, blood cell counts, and routine biochemical parameters, supporting robustness across diverse clinical settings.

## 1. Introduction

The development of liquid biopsy-based RNA diagnostic tools represents a major advance in precision medicine, enabling minimally invasive assessment of disease-associated molecular signatures. Circulating RNA species—including messenger RNA (mRNA), microRNA (miRNA), and other non-coding RNAs—are detectable in whole blood, plasma, and serum and reflect dynamic physiological and pathological processes across multiple organ systems [1,2,3,4]. The advantage of peripheral blood sampling over tissue biopsy for serial monitoring and minimally invasive disease assessment is well-established in the liquid biopsy literature [5,6].

Despite this promise, the clinical implementation of blood-based RNA diagnostics requires rigorous analytical validation to ensure reliability, reproducibility, and regulatory compliance [7]. Pre-analytical variables—including blood collection tubes, anticoagulants, processing time, storage conditions, and RNA extraction methods—can significantly influence RNA yield, integrity, and expression profiles [8,9,10]. In addition, low RNA abundance, susceptibility to degradation, and biological variability introduce analytical challenges that must be systematically characterized. Without careful control and documentation of these factors, assay performance may be compromised, limiting clinical utility [10].

Analytical validation establishes the technical performance characteristics of an assay, including accuracy, precision, limit of detection (LoD), limit of quantification (LoQ), linearity, analytical specificity, and robustness. Regulatory and professional guidelines emphasize the importance of defining these parameters prior to clinical deployment (e.g., CLSI guidelines: EP17 [LOD/LOQ], EP05 [precision]). For PCR-based RNA expression assays in particular, reproducibility across operators, instruments, reagent lots, and testing sites is critical, as inter-laboratory variability can meaningfully affect diagnostic classification [11].

In this study, we present the analytical validation of a novel blood-based RNA diagnostic tool (NETest2.0^®^) designed to quantify disease-associated gene expression signatures from peripheral blood specimens from patients with neuroendocrine tumors (NETs) [12]. As published elsewhere [12], the raw PCR data—cycle threshold (Ct)—was fed into a proprietary machine learning (ML) algorithm to transform the raw data from 51 cancer genes and 4 housekeeping genes (HKGs) into a score on a scale of 0–100. A medical cut-off of 50 (≥50 = NET and <50 = No NET) was established and validated in a clinical court [12]. We systematically evaluated pre-analytical and analytical performance characteristics, including assay precision, analytical sensitivity, and interference testing. The results provide evidence supporting the reliability and fitness of NETest2.0^®^ for clinical application, establishing a technical foundation for subsequent clinical validation and utility studies.

## 2. Materials and Methods

Approach: Table 1 summarizes the different validation studies undertaken. These are included in the following CLSI guidelines: EP05, EP06, EP07, EP17, and MM17 [13]. Transportation studies included ISTA 7D 2007 “Summer Profile” and “Winter Profile”.

Assay: NETest2.0^®^ is a 55-marker gene assay derived from peripheral blood collected into RNA stabilization tubes [12]. The 55-gene assay includes 51 NET markers and 4 housekeeping genes. The marker set includes biologically validated genes involved in fundamental hallmarks of neuroendocrine neoplasia, including proliferation, secretion, metabolism, signaling, angiogenesis, invasion, epigenetic regulation, and tumor microenvironment interactions [14,15]. The 4 housekeeping genes were selected based on stable expression across NET and non-NET samples [16]. The final gene set was selected through prior optimization studies (including wet-lab work and literature review and curation) as well as algorithmic training procedures.

Total RNA is isolated using the RNeasy MiniKit (Qiagen, Valencia, Santa Clarita, CA, USA; RNA quality ≥ 1.8 A_260:280_ ratio, mRNA production: 2.6–34 µg/sample) [16]. Complementary DNA is synthesized using the High-Capacity cDNA Reverse Transcriptase Kit (Thermo Fisher Scientific, Waltham, MA, USA; typical cDNA yield 2000–2500 ng/µL) and real-time PCR performed in pre-spotted TaqMan PCR primer plates using 200 ng/µL of cDNA/well [17]. PCR is run on a QuantStudio Flex 7 instrument (Thermo Fisher Scientific). The pre-spotted TaqMan primer plates and PCR master mix are custom-manufactured for NETest2.0^®^ by ThermoFisher Scientific under controlled production specifications and supplied in a standardized ready-to-use kit format. Each production lot undergoes manufacturer quality control testing, including verification of amplification efficiency, specificity, and absence of non-specific signal prior to release. Reagent lot traceability and batch documentation are maintained within the laboratory quality system to ensure consistency across testing periods and to minimize inter-lot variability. A positive control (derived from a 1:1 mix of two NET cells lines, H720 and H727) is included on each plate.

**Figure 1:**
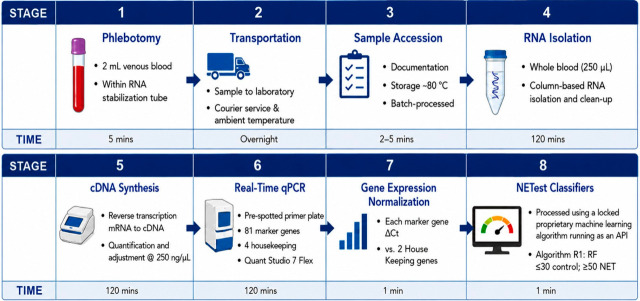
Overview of the NETest2.0^®^ workflow. RF = random forest.

Target (*n* = 51 NET) transcript levels were normalized to 4 different housekeeping genes—*ALG9*, *ATG4B*, *RHOA*, and *TXNIP*—and quantified using the ΔΔCt [16]. The RT-PCR data were processed through the NETest2.0^®^ algorithm [12]. Results were expressed as a risk stratification score (0–100) for NET, which was converted into a binary readout (positive/negative). A cut-off score ≥ 50 was used to detect a neuroendocrine tumor (sample = positive). The step-by-step process is included in Figure 1.

Blood samples: All samples were collected either on the day of imaging or within 30 days. Samples were collected into proprietary RNA stabilization tubes (Nuclisafe™ Wren stabilization buffer) validated to preserve RNA integrity for 7–10 days at ambient temperature (internal validation data). Across validation experiments, the assay failure rate was <0.1%, reflecting stable extraction and amplification performance. For accuracy, batch processing was performed.

Accuracy: This included 568 NETs (all that had histological and image-based confirmation of disease, grade and stage are included—Table 2) and 219 other histologically confirmed cancers from different gastrointestinal [GI] sites (*n* = 79), pancreas (*n* = 23), lung (*n* = 59), breast (*n* = 12), prostate (*n* = 39) and others (*n* = 7, including a GIST [gastrointestinal stromal tumor], 3 RCCs [renal clear cell carcinomas] and 3 melanomas). In addition, 186 controls were also evaluated. We also evaluated different cut-off points for the algorithm to assess accuracy in these cohorts. NETest2.0^®^ scores were compared to imaging and biopsy results as ground truth for NET disease detection. We thereafter evaluated whether grade or stage impacted NETest2.0^®^ scores in the 568 NETs. For these studies, we evaluated the AUC and diagnostic metrics vs. 219 non-NET samples and 186 controls (*n* = 405).

Precision studies: These included both contrived blood (normal blood samples spiked with NCI-H727cells [18]) and neat NET clinical samples (*n* = 6, 1 negative and 5 positive samples as confirmed by imaging and biopsy; Table 3).

For the blood spike-in studies, the intra- and inter-assay variability was assessed using the positive control NCI-H727 cell line (1000 cells/mL) spiked directly into control blood samples. PCR was run in triplicate and over 9 separate days. For clinical samples, PCR was run by two different operators on two different PCR instruments over a 5-day period that was repeated over 4 weeks (total = 20 days of PCR). For intra-assay evaluations, 2 samples were included on a plate. Each operator ran 6 plates per day (i.e., each of the clinical samples were run by both operators each day for 10 days). For inter-assay evaluations, 3 different samples were included on a plate and repeated (*n* = 2 plates/3 different samples). Each operator ran 4 plates per day (i.e., each of the 6 clinical samples were repeated by both operators each day for 10 days). The operators switched between 2 PCR instruments to ensure assays were evaluated on both Flex7 instruments. Two different reagent lots were used. Ct values (averaged over the 55 genes) and NETest2.0^®^ scores were evaluated.

Limit of Blank, Limit of Detection and Limit of Quantification Studies: LoB, LoD and LoQ were evaluated by spiking the NCI-H727 cell line into stabilization buffer [18]. Serial dilutions were made from 10 cells/mL to 0.1 cells/mL (3 dilutions). RNA was extracted (5 different experiments). PCR was run on samples in quadruplicate (*n* = 20 PCR runs in total, per dilution). Ct and NETest2.0^®^ scores were evaluated for the 3 dilutions. Calculations for LoD and LoQ were performed using averaged Ct values across target genes [19] and were confirmed using algorithm-derived NETest2.0^®^ score outputs.

Analytical Specificity studies: Standard interfering substance studies were undertaken. For exogenous interfering substances, we evaluated spiked blood exposed to one of the following commonly prescribed agents: aspirin (60 mg/dL), acetaminophen (45 mg/dL), ibuprofen (20 mg/dL), metformin (45 mg/dL) or omeprazole (6 mg/dL). For endogenous interfering substances, we evaluated spiked blood exposed to hemoglobin (25 g/dL to simulate severe hemolysis) or triglycerides (500 mg/dL), bilirubin (2.5 mg/dL), urea (25 ng/dL) or glucose (130 mg/dL). All chemicals for the interfering substance studies were obtained from Sigma Chemical Company (St. Louis, MO, USA). Individual chemicals were dissolved in the appropriate solvent and added to individual aliquots (1 mL) of normal whole blood spiked with 10,000 H727 cells to meet the final pathophysiological dilution. We also evaluated whether NETest2.0^®^ scores were affected by age, gender, or ethnicity (Table 4).

Furthermore, we examined the relationship between standard clinical blood counts and NETest2.0^®^ scores to evaluate whether scores were affected by differences in common circulating blood cell populations. Additionally, we examined whether NETest2.0^®^ scores correlated with standard clinical factors, e.g., LDH levels or kidney function (e.g., glomerular filtration rate) or liver function (e.g., ASAT/AST or ALAT/ALT levels). This was undertaken in 130 NET patients. All hematological and blood biochemistry parameters were assessed per standard clinical laboratory testing.

Linearity Range studies: cDNA obtained from the NCI-H727 cell line [18] was generated (measured at 2000 ng/uL). Serial dilutions were made at the following dilutions: 0.01 ng/µL to 200 ng/µL (final concentration of cell line-derived cDNA). PCR was run on samples in quadruplicate. Ct and NETest2.0^®^ scores were evaluated for each of the dilutions’ Ct values across the target genes [19] and algorithm-derived NETest2.0^®^ scores were evaluated.

Robustness and Stability studies: Ambient and freezer stability as well as transportation studies were undertaken.

Ambient storage: Stability at ambient temperature (22 °C) was evaluated for whole blood in Wren stabilization buffer tubes. Ten NET and 5 control samples were evaluated over a 21-day period. In all studies, RNA isolation, cDNA synthesis and PCR were run by two different operators on two different PCR instruments. mRNA RIN values, Ct values and NETest2.0^®^ scores were compared to those of samples collected and stored immediately.

Storage (−80 °C Freezer): Long-term stability was evaluated for stored RNA-stabilized whole blood (−80 °C). Clinical samples (stored over a 5-year period: 2019–2023) were evaluated. Three NET and 2 control samples were evaluated per year (total = 25 samples). In all studies, RNA isolation, cDNA synthesis and PCR were run by two different operators on two different PCR instruments. Studies were undertaken over a 5-day period. mRNA RIN values were obtained for re-isolated samples. Both C_t_ values and NETest2.0^®^ scores were compared to original sample scores (in the year they were processed).

Shipping: The International Safe Transit Association (ISTA) Test procedure 7D published in 2007 (ISTA 7D 2007—Temperature Test for Transport Packing [20]) “Summer Profile” and “Winter Profile” were followed to evaluate shipping stability. For “Summer”, temperatures are changed between +22 °C and +40 °C over a 56 h period. For “Winter”, temperatures are changed between −10 °C and +18 °C (over 56 h). Aliquots were collected for bioanalyzer measurements and PCR performed. Thirty NET and 30 control samples were evaluated. In all studies, isolations and PCR were undertaken by two different operators on two different PCR instruments. mRNA RIN, C_t_ values and NETest2.0^®^ scores were compared to original sample scores (pre-stability evaluation).

Data analyses and Statistics: Raw Ct values as well as normalized values and NETest2.0^®^ scores were calculated using the ML algorithm. Results were expressed as a risk stratification score (0–100) for NET, which was converted into a binary readout (positive/negative). AUROC analyses and diagnostic metrics were calculated for the accuracy studies. The 95% confidence intervals were included for AUCs. Diagnostic metrics included sensitivity, specificity, positive predictive value (PPV), negative predictive value (NPV), and overall accuracy. PROBIT analyses were undertaken for LoD and LoQ. For precision studies, individual Ct levels as well as averaged Ct levels for each of the individual 55 genes across the experiments were evaluated. Non-parametric Mann–Whitney, ANOVA (Kruskal–Wallis) and Pearson correlations were calculated. All analyses were performed using Prism version 9.4 for Windows (GraphPad Software Inc., La Jolla, CA, USA, www.graphpad.com, accessed on 1 April 2026) and MedCalc Statistical Software v23.2.1 (MedCalc Software Ltd., Ostend, Belgium; http://www.medcalc.org; 2017, accessed on 30 March 2026). Statistical significance was defined as *p* < 0.05.

## 3. Results

**Figure 2:**
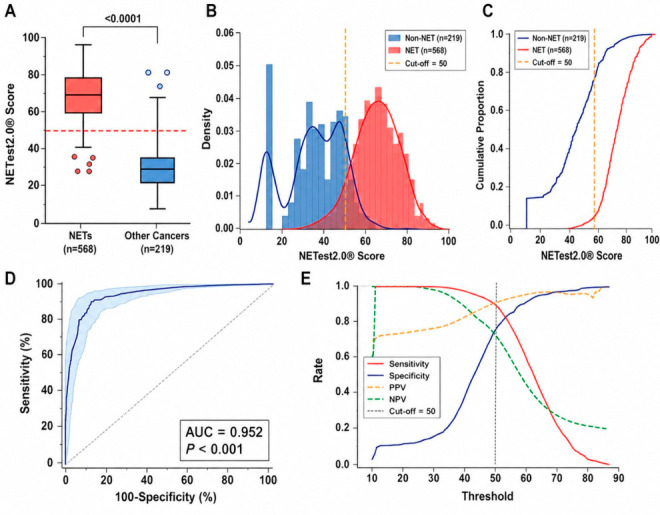
NETest2.0^®^ scores in 787 subjects including 568 NETs and 219 cancer (non-NET) subjects. (**A**). Individual NETest2.0^®^ scores in NETs and other cancers (non-NETs). Scores were significantly higher in NETs. (**B**). Distribution of scores in cancers (blue) and NETs (red). (**C**). Cumulative scores in cancers (blue) and NETs (red). (**D**). AUROC analysis for NET vs. cancers. The 95% CI is included. (**E**). Line graphs for sensitivity, specificity, PPV and NPV at different thresholds in the 787 cohort. Abbreviations: AUC = area under the curve, NPV = negative predictive value, PPV = positive predictive value. Dotted lines (red/orange) identify the cut-off score of 50. Mean ± SD.

1. Accuracy and Sensitivity: We initially compared NETest2.0^®^ scores in 568 patients with image-confirmed NET disease and compared them to 219 non-NET malignancies (epithelial cancers). NETest2.0^®^ was positive in 520 (91.5%) of NETs and in 23 (10.5%) of cancers (*p* < 0.0001, Figure 2A). The distribution of NETest2.0^®^ scores is included in Figure 2B and the cumulative distribution in Figure 2C. The AUROC analysis identified an AUC of 0.95 ± 0.01 (95%CI: 0.935–0.966) with an associated cut-off of 49.5 (on the 0–100 scale, Figure 2D). Evaluations of different thresholds (Figure 2E) confirmed the standard cut-off [12] of 50 to be valid to differentiate between a “NET” and “non-NET”. The diagnostic metrics were sensitivity 91.5%, specificity 89.5%, PPV 95.8%, NPV 80.3% and overall accuracy 91.0%.

**Figure 3:**
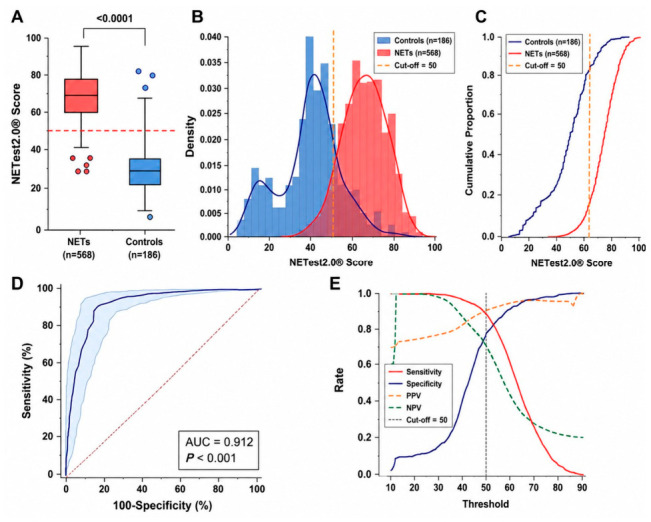
NETest2.0^®^ scores in 756 subjects including 568 NETs and 186 control subjects. (**A**). Individual NETest2.0^®^ scores in NETs and controls. Scores were significantly higher in NETs. (**B**). Distribution of scores in controls (blue) and NETs (red). (**C**). Cumulative scores in controls (blue) and NETs (red). (**D**). AUROC analysis for NET vs. controls. The 95% CI is included. (**E**). Line graphs for sensitivity, specificity, PPV and NPV at different thresholds in the 756 cohort. Abbreviations: AUC = area under the curve, NPV = negative predictive value, PPV = positive predictive value. Dotted lines (red/orange) identify the cut-off score of 50. Mean ± SD.

We then compared NETest2.0^®^ scores in 568 patients with NET disease and compared them to 186 controls. NETest2.0^®^ was positive in 35 (18.9%) of controls (*p* < 0.0001, Figure 3A). The distribution of NETest2.0^®^ scores is included in Figure 3B and the cumulative distribution in Figure 3C. The AUROC analysis identified an AUC of 0.91 ± 0.01 (95%CI: 0.09–0.93) with an associated cut-off of >49 (on the 0–100 scale, Figure 3D). Evaluations of different thresholds (Figure 3E) confirmed the standard cut-off of 50 to be valid to differentiate between a “NET” and “control”. The diagnostic metrics using a cut-off of 50 were sensitivity 92.4%, specificity 81.2%, PPV 93.7%, NPV 77.8% and overall accuracy 89.6%.

**Figure 4:**
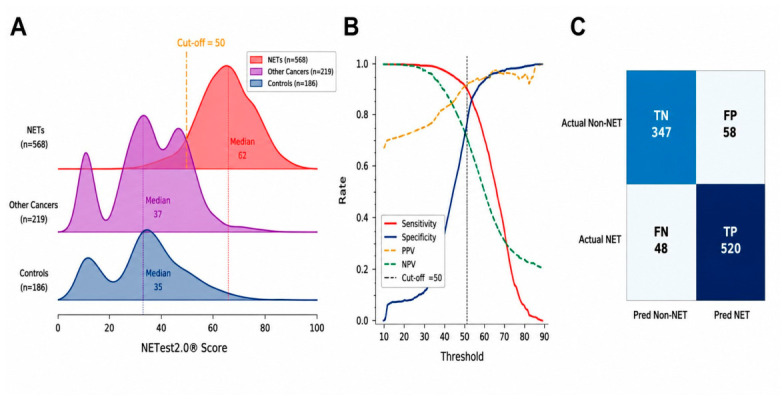
NETest2.0^®^ scores in NETs vs. other cancers and control subjects. (**A**). Distribution of scores in controls (blue) and NETs (red). (**B**). Line graphs for sensitivity, specificity, PPV and NPV at different thresholds in the 973 cohort. (**C**). A 2 × 2 table for NETs (*n* = 568) vs. non-NETs (*n* = 405). Abbreviations: NPV = negative predictive value, PPV = positive predictive value.

The distribution of NETest2.0^®^ scores in NETs compared to all non-NETs (other cancers and controls) demonstrated minimal overlap (Figure 4A). Thresholds for NETs vs. all others are included in Figure 4B, identifying 50 as an appropriate cut-off. The 2 × 2 table is included in Figure 4C. Diagnostic metrics using a cut-off of 50 (from the 2 × 2 table [Figure 4C]) were: sensitivity 91.6%, specificity 85.7%, PPV 90.0%, NPV 87.9% and overall accuracy 89.1%.

**Figure 5:**
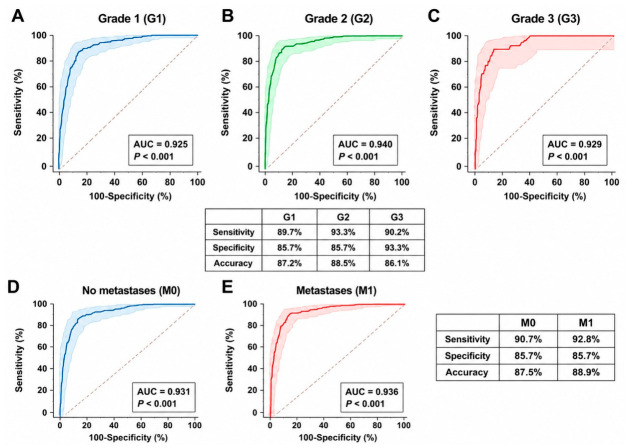
Assessment of NETest2.0^®^ (positive/negative) for detecting NET disease based on grade and stage. (**A**–**C**) AUCs for each of the three grades, G1, G2 and G3 tumors, and the metrics for sensitivity, specificity and overall accuracy. (**D**,**E**) AUCs for each of the stages (M0: I–III and M1: IV) are included as are the metrics for sensitivity, specificity and overall accuracy. Abbreviations: AUC = area under the curve.

In a sub-analysis of the 568 NETs, we evaluated whether grade or stage impacted NETest2.0^®^ scores. For these studies, we compared the AUC and diagnostic metrics vs. non-NET/control (*n* = 405) samples. Tumor grade was available in 534 NETs (94.0%). AUCs for each of the three grade types ranged from 0.925 ± 0.01 in G1 to 0.94 ± 0.01 in G2 and 0.93 ± 0.02 in G3 (Figure 5A–C). The diagnostic metrics for the NETest2.0^®^ by grade are included. Sensitivities ranged from 89.7 to 93.3%, specificity was 85.7% and accuracies were 86.1–88.5%. Stage was available in all cases. Two hundred and thirty six (41.5%) were Stage I–III and 332 (58.5%) were Stage IV (liver metastases). The AUCs were 0.93 ± 0.01 and 0.94 ± 0.01, respectively (Figure 5D,E). Sensitivities were 90.7 and 92.8%, specificity was 85.7% and accuracies were 87.5–88.9%.

2. Precision: We examined both cell line spike-in samples and clinical samples.

**Figure 6:**
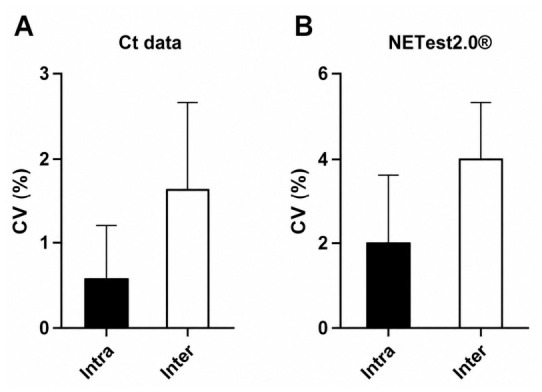
Evaluation of variability (Ct and scores). (**A**). Intra- and inter-assay variability (coefficient of variation) for Ct values. (**B**). Intra- and inter-assay variabilities for NETest2.0^®^ scores. Mean ± SD.

2a. Positive control spike-in: For intra-assay variability, we evaluated Ct values as well as the NETest2.0^®^ score generated from spike-in samples. The intra-assay variation for Ct values was 0.56 ± 0.56% (Figure 6A). The coefficient of variability for NETest2.0^®^ score was 2.1 ± 1.6% (Figure 6B). The inter-assay variability for Ct values was 1.7 ± 0.9% (Figure 6A). The coefficient of variability for the NETest2.0^®^ score was 4.1 ± 1.2% (Figure 6B).

**Figure 7:**
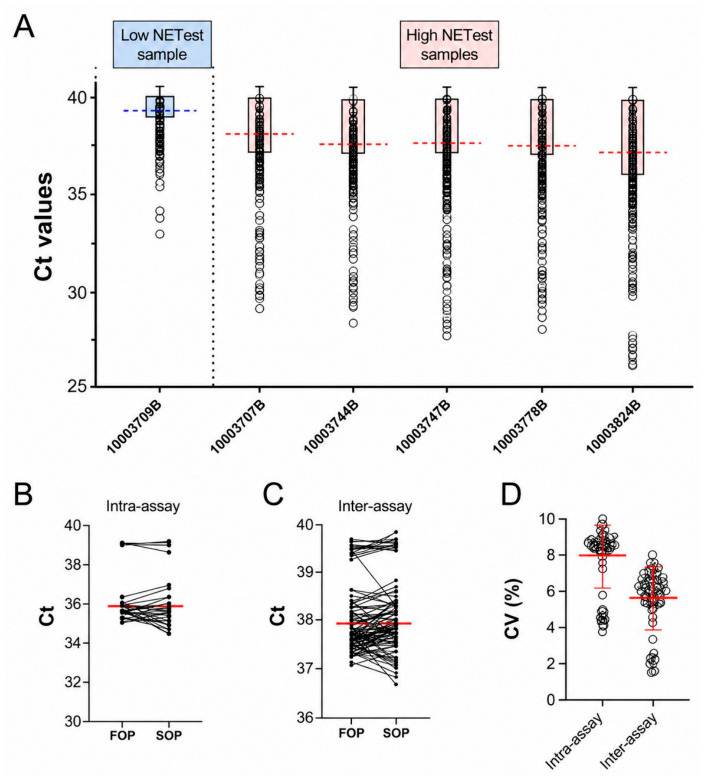
Evaluation of Ct levels (individual (**A**), averaged for each of the individual 55 genes across the experiments (**B**,**C**)) in the 6 clinical samples. (**A**). Individual Ct values by operator (operator 1 or operator 2) and by sample type. Values were not different between the two operators. (**B**). Intra-assay reproducibility between the first operator (FOP) and second operator (SOP). The red line is the mean value. (**C**). Inter-assay reproducibility between the FOP and SOP. The red line is the mean value. (**D**). Intra- and inter-assay coefficient of variation (CV). For (**A**): red line = mean. Boxes represent the interquartile ranges (25–75%). Blue = low NETest2.0^®^ score sample; red = high NETest2.0^®^ score samples. Individual Ct values are plotted (aligned plot). For (**B**–**D**): mean ± SD.

2b. Clinical NET samples: An overview of Ct values from each of the two operators over the 20-day period is included in Figure 7A. An evaluation of Ct values in the intra-assay studies identified no significant difference in mean Ct values (first operator [FOP]: 36 ± 1.4 vs. second operator [SOP]: 36 ± 1.5, *p* = 0.18, Mann–Whitney test; Figure 7B). Ct values were well-correlated (Pearson r = 0.94 [95%CI: 0.88–0.97], *p* < 0.0001). In the inter-assay evaluation, Ct values were similarly not different (FOP: 38 ± 0.75 vs. SOP: 38 ± 0.78, *p* = 0.189, Mann–Whitney test; Figure 7C). Ct values were well-correlated (Pearson r = 0.89 [95%CI: 0.85–093]), *p* < 0.0001). The calculated intra- and inter-assay variations for Ct values were 8.1 ± 1.7% and 5.6 ± 1.6%, respectively (Figure 7D). An evaluation of the individual housekeeping genes identified stable expression and minimal variability across the tested conditions: *ALG9*: 37.4 ± 0.8, CV% = 2.3; *ATG4B*: 33.5 ± 0.7, CV% = 2.1; *RHOA*: 29.2 ± 0.7, CV% = 2.3; and *TXNIP*: 28.2 ± 0.6, CV% = 2.3.

**Figure 8:**
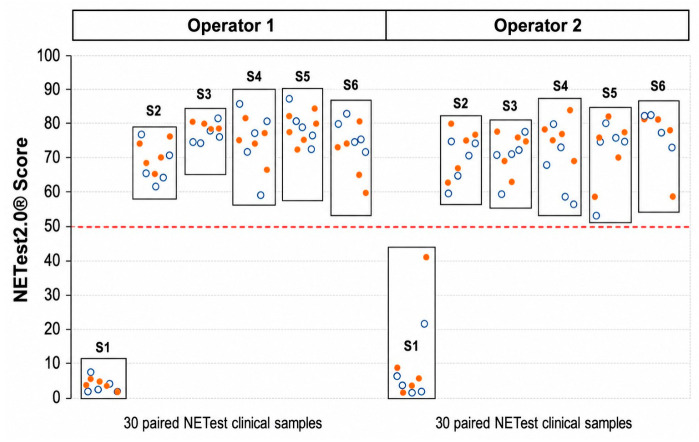
Precision of NETest2.0^®^ scores. NETest2.0^®^ scores by operator and sample (*n* = 30 pairs [6 samples/5 repeats] per operator). The individual samples (S1–6) are boxed. First sample is blue empty circle, orange dot is the second sample in the pair, The horizontal dashed red line is the upper limit of normal.

2c. NETest2.0^®^ clinical scores:

Precision: NETest2.0^®^ score precision is included in Figure 8. One NET-negative and five NET-positive samples were used in the precision study. With a qualitative (binary readout) assessment, all 20 replicates from the negative sample tested negative and all 100 replicates from the five positive samples tested positive. For the numeric scores, excluding one outlier, CV% between replicates of each of the six samples ranged from 3.5 to 9.7%.

Intra-assay precision: An evaluation of the intra-assay metrics (between the two replicates) identified that all 20 replicates from the negative sample tested negative and all 100 replicates from the five positive samples tested positive. With the exclusion of one outlier out of the 60 paired readouts, for the numeric scores (*n* = 59), CV% was 5.6 ± 4.5%.

An evaluation of these NETest2.0^®^ scores identified no significant difference in mean scores between the operators (FOP: 54 ± 26 vs. SOP: 57 ± 23, *p* = 0.64, Mann–Whitney test); scores were well-correlated (Pearson r = 0.82, *p* < 0.0001). In the inter-assay evaluation, NETest2.0^®^ scores were similarly not different (FOP: 64 ± 29 vs. SOP: 65 ± 28, *p* = 0.98, Mann–Whitney test) and were well-correlated (Pearson r = 0.94, *p* < 0.0001). An evaluation of NETest2.0^®^ scores by day identified no differences in scores over the time-period evaluated (*p* = 0.905, Welch’s ANOVA), while an evaluation of NETest2.0^®^ scores by PCR instrument identified no differences in scores (Table 5).

**Figure 9:**
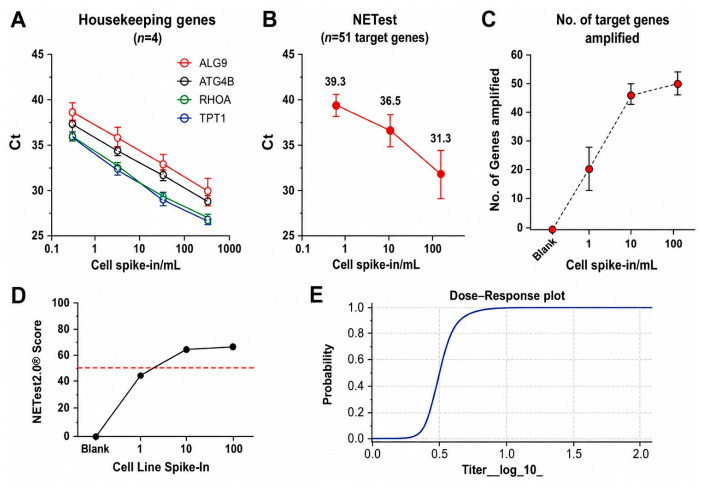
Evaluation of limits of blank, detection and quantification in spiked-in blood and examination of NETest2.0^®^ scores and PROBIT calculation. (**A**). Housekeeping gene expression (Ct values) as a function of cell number (spike-in). (**B**). NETest2.0^®^ target marker gene expression (Ct values) as a function of cell number. The numbers reflect the mean Ct values for each of the cell numbers/mL. (**C**). Number of genes amplified as a function of cell number. (**D**). NETest2.0^®^ scores as a function of cell spike-in. Positive scores were identified from 1 to 100 cells/mL. (**E**). PROBIT analysis of NETest2.0^®^ scores. The calculated LOD (95% detectable scores) = 2 cells/mL. Mean ± SD. The red line in (**D**) is the cut-off point/upper limit of normality for the NETest2.0^®^ score.

**Figure 10:**
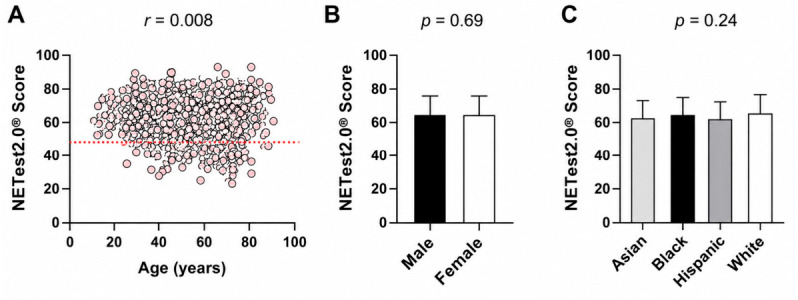
Assessment of the relationship between age, sex and ethnicity for the NETest2.0^®^ score. (**A**). No correlation was noted between age and score (line = linear correlation curve). (**B**). Sex/gender had no significant effect on the score (*p* = 0.69, 2-tailed Mann–Whitney test). (**C**). Ethnicity was not related to the score (*p* = 0.24, 2-tailed, Mann–Whitney test). The red line in (**A**) is the cut-off point/upper limit of normality for the NETest2.0^®^ score. Mean ± SD.

3. Limit of Blank, Detection and Quantification: All 51 target transcripts were amplified in cell line-derived RNA (Ct < 35; mean PCR efficiency 1.94 ± 0.12). Housekeeping gene expression was detectable (Ct < 40) with as little as 1 cell/mL of buffer (Figure 9A). Target gene expression was also detectable with as little as 1 cell/mL (Figure 9B). The number of target genes amplified ranged from 20 ± 6 (at 1 cell/mL) to 50 ± 0.5 (100 cells/mL; Figure 9C). The calculated LoB was 0 (NETest2.0^®^ score 0, no cells spiked-in). The calculated LoD from the Ct values (LoD = [3.3 × (SD/slope)]) was 2.2 cells/mL, while the calculated LoQ from the Ct values (LOQ = [10 × (SD/slope)] was 8.6 cells/mL. NETest2.0^®^ scores were positive between 1 and 100 cells/mL (Figure 9D). PROBIT analyses (Figure 9E) of the NETest2.0^®^ score identified that the limit of detection (95% probability of detecting a positive score) was 10^0.358^ = 2.29 cells/mL.

4. Analytical Specificity: We examined both general factors as well as interfering substances:

4a. General factors: No significant differences were noted in age, gender, and ethnicity for NETest2.0^®^ scores (Figure 10).

**Figure 11:**
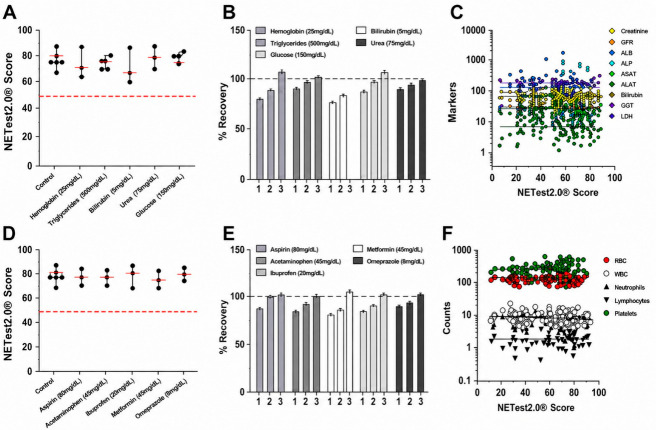
Relationship between endogenous, exogenous and blood factors on NETest2.0^®^ scores. (**A**). Endogenous substances spiked into whole blood samples did not impact NETest2.0^®^ scores (Kruskal–Wallis statistic: 1.724, *p* = 0.856). (**B**). Percentage recovery was −3.94% for endogenous substances. (**C**). No significant correlation was identified between markers of kidney function and hepatic function and NETest2.0^®^ output. (**D**). Exogenous substances spiked into whole blood samples did not impact NETest2.0^®^ scores (Kruskal–Wallis statistic: 2.302, *p* = 0.806). (**E**). Percentage recovery was −1.65% for endogenous substances. (**F**). No significant correlation was identified between blood cell numbers and NETest2.0^®^ output. Kidney function (Creatinine, GFR = glomerular filtration rate) and liver function (LDH = lactate dehydrogenase, ALP = alkaline phosphatase, GGT = gamma-glutamyl transferase, ALB = albumin). RBC = red blood cells, WBC = white blood cells. The red line in (**A**,**D**) is the cut-off point/upper limit of normality for the NETest2.0^®^ score.

4b. Interfering substances: Endogenous factors: We examined this in two ways. Firstly, we used spike-in studies to examine whether specific factors, e.g., hemoglobin, affected NETest2.0^®^ scores. All factors evaluated had no significant effect on scores (Figure 11A, *p* = 0.86) and the overall recovery was −3.94% (Figure 11B). No relationship was identified between different measures of kidney function, e.g., GFR or hepatic function (e.g., Bilirubin levels) and NETest2.0® scores (Figure 11C). The non-significant correlations ranged between −0.073 (ASAT) and +0.057 (ALP). Secondly, we evaluated the impact of exogenous factors on NETest2.0^®^ scores. All factors evaluated had no significant effect on NETest2.0^®^ scores (Figure 11D, *p* = 0.81) and the overall recovery was −1.65% (Figure 11E). Finally, we evaluated whether NETest2.0^®^ scores were related to blood cell counts, e.g., lymphocytes, neutrophils or platelets. In a group of *n* = 130 NETs, no correlation was identified between NETest2.0^®^ score (output) and nucleated or non-nucleated (red blood cell) numbers. R-correlations ranged between −0.21 (neutrophils) and +0.04 (platelets) (Figure 11F).

5. Linearity and Range: We examined this using cell lines spiked into blood samples.

**Figure 12:**
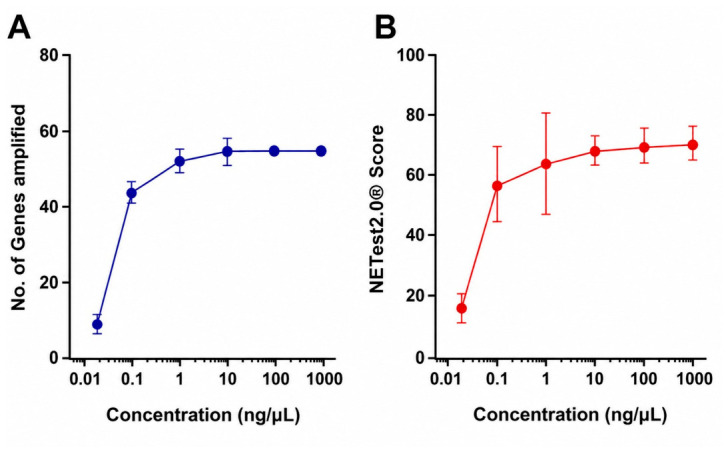
Linearity and range. (**A**). Number of genes detected between 0.01 and 200 ng/µL cDNA spiked into blood. (**B**). NETest2.0^®^ scores derived from spike-ins of 0.01–200 ng/µL cDNA. Mean ± SD (*n* = 6). The red line in (**B**) is the cut-off point/upper limit of normality for the NETest2.0^®^ score.

Cell line studies for LoD/LoQ identified that increasing concentrations of cell line > 100 per mL resulted in a plateau of NETest2.0^®^ scores (see Figure 9D). The spike-in studies utilized ~1 ng/µL cDNA per PCR reaction. In these studies, we evaluated concentration ranges from 0.01 ng/µL cDNA to 200 ng/µL cDNA. The 51 marker genes were consistently amplified from 1 ng/µL to 200 ng/µL (Figure 12A) and consistent positive scores were noted between 1 and 200 ng/µL spike-in (Figure 12B).

6. Robustness and Stability: We examined robustness by evaluating sample buffer stability at ambient temperature and sample integrity after storage for 5 years at −80 °C. In a second set of experiments, we examined transportation stability evaluating the impacts of both “Summer” and “Winter” transport conditions.

**Figure 13:**
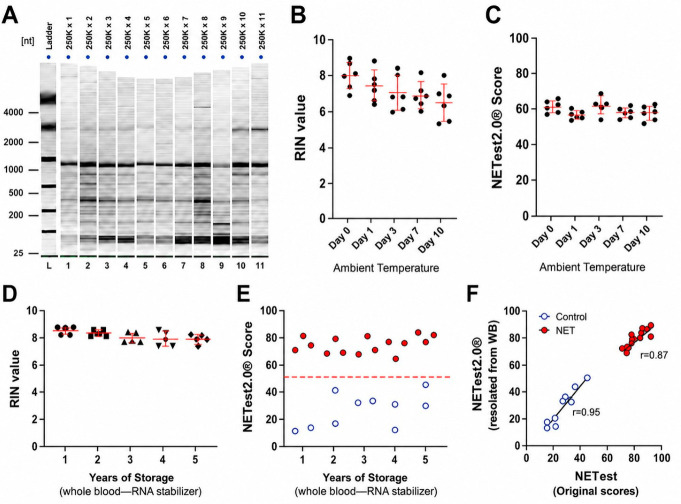
Buffer stability and storage robustness. (**A**). Bioanalyzer electropherogram data from ambient-stored samples. Typical 18S and 28S bands are visible. All samples had RIN values of 5.9–7.7 in this example. (**B**). Individual RIN values from 6 samples stored for 0–10 days at ambient temperature. (**C**). NETest2.0^®^ scores from the same 6 samples stored for 0–10 days at ambient temperature. (**D**). RIN values from 5 clinical samples stored for 0–5 years at −80 °C. (**E**). Individual scores from the 5 samples (3 NETs—red, 2 controls—blue) over the 5-year period. (**F**). Correlation between scores at time 0 (original score) and at subsequent follow-up time points. Scores were highly concordant. Abbreviations: RIN = RNA integrity number; WB = whole blood. The red line in (**E**) is the cut-off point/upper limit of normality for the NETest2.0^®^ score.

6a. Ambient stability: We evaluated buffer stability at ambient temperature (22 °C) for 0–10 days measuring impacts on RIN values (degradation) and NETest2.0^®^ scores. Figure 13A includes data from the bioanalyzer demonstrating RNA integrity. This is confirmed in Figure 13B. RIN values were >5 across all 10 days. No significant differences were noted between Day 0 and any of the follow-up time points (Kruskal–Wallis ANOVA: 6.25, *p* = 0.181). This RNA stability translated into stable NETest2.0^®^ scores over the 10-day period (Figure 13C, Kruskal–Wallis ANOVA: 8.97, *p* = 0.06).

6b. Freezer stability: We evaluated sample storage stability at −80 °C for 0–5 years evaluating RIN values and scores. No significant differences were identified in RIN values over the 5 years of sample storage (Figure 13D; Kruskal–Wallis ANOVA: 8.22, *p* = 0.08). Three negative and three positive NETest2.0^®^ samples exhibited concordant scores across the storage time (Figure 13E); scores were highly correlated (Figure 13F).

**Figure 14:**
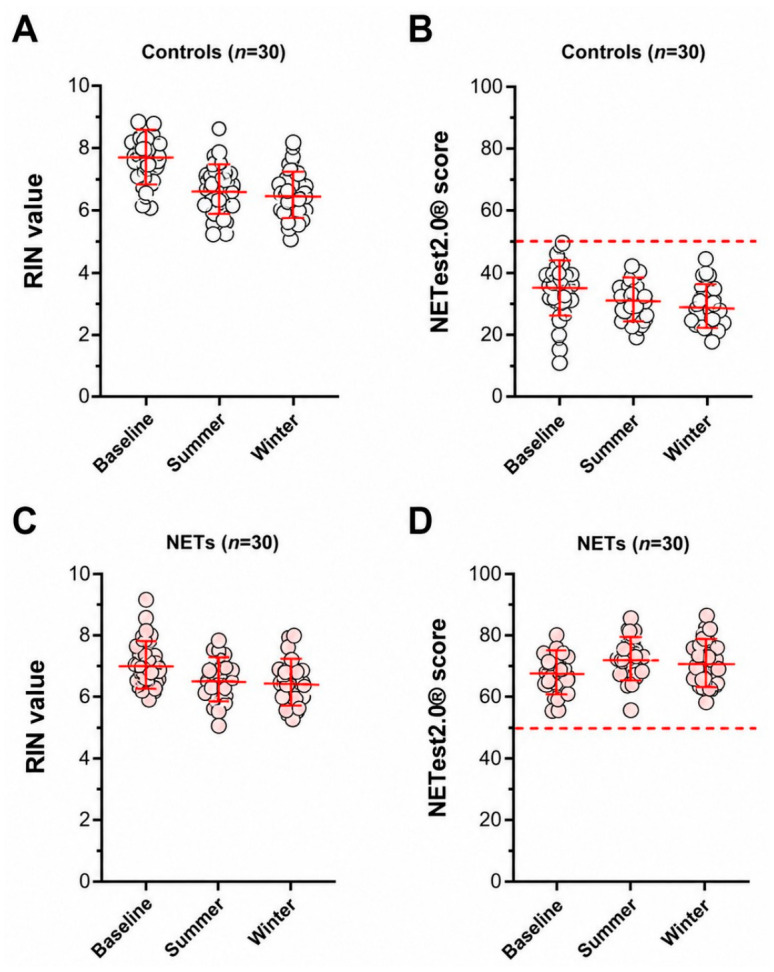
RNA integrity and NETest2.0^®^ scores as a function of transportation. (**A**). Bioanalyzer results for 30 control samples identified no significant degradation by summer or winter conditions. (**B**). NETest2.0^®^ scores for controls identified no impact of summer/winter transportation. (**C**). Bioanalyzer results identified no impact of summer or winter conditions on RNA integrity in 30 NETs. (**D**). NETest2.0^®^ scores for NETs under summer and winter transportation conditions. Mean ± SD. The red dotted line is the cut-off of 50 for NETest2.0^®^.

6c. Transportation stability: No differences were identified in either the RIN values in controls (Figure 14A) or NETest2.0^®^ scores (Figure 14B). Similarly, neither transportation temperature profiles affected RIN (Figure 14C) or NETest2.0^®^ scores (Figure 14D) in the 30 NET samples.

## 4. Discussion

This study was designed to evaluate analytical validity and assay performance characteristics of NETest2.0^®^, a multigene qPCR assay designed to quantify neuroendocrine tumor-associated transcripts in peripheral blood rather than clinical utility or patient management outcomes.

The validation framework was structured in accordance with established CLSI principles for molecular assay evaluation, including EP17 (limit of detection and quantification), EP05 (precision and reproducibility), and EP06 (linearity and measurement range). The results demonstrate that the assay meets accepted analytical performance thresholds for sensitivity, precision, and robustness, supporting its suitability for standardized clinical laboratory implementation. Future studies including multiple sites and expanded external validation cohorts will help further define inter-laboratory reproducibility, workflow standardization, and performance across geographically diverse clinical settings.

NETest2.0^®^ exhibited high diagnostic performance in distinguishing NETs from non-NET malignancies and non-cancer controls, achieving sensitivities of 91.5—92.4% and specificities of 81.2—89.5%, with an AUC of 0.91—0.95. These metrics compare favorably with existing NET biomarkers, which are often limited by suboptimal sensitivity or specificity. Importantly, the high positive predictive values (93.7—95.8%) suggest that a positive NETest2.0^®^ score is strongly indicative of NET disease, while the lower negative predictive value (77.8—80.3%) reflects the inherent challenge of excluding disease in heterogeneous tumor populations. Samples were collected at the time of imaging or within 30 days. This was undertaken to minimize any potential discordance between scores and imaging results due to interval disease progression or treatment effects but may have an impact on the PPV and NPV.

The use of a standardized cut-off (score ≥ 50) was validated across multiple threshold analyses and in both control populations, demonstrating stability and robustness of classification. The consistent accuracy across grade (G1—G3) and all stages (Stage I–III to IV) supports the accuracy of the assay. This is particularly relevant in clinical practice, where threshold reproducibility is essential for consistent interpretation. The clear separation of score distributions between NET and non-NET cancers/controls further supports the biological specificity of the assay and highlights the advantage of a multigene transcriptional signature over single-analyte biomarkers. Regarding the NETs that were NETest2.0^®^-negative, several explanations exist including low proliferative activity, necrotic or fibrotic disease, and limited vascular access but the most likely explanation is transcriptional silencing perhaps as a result of successful therapy. A previous study identified that serial changes (decreases) in NETest2.0^®^ score including scores that are negative (<50) were associated with disease stabilization and response to therapy [21].

Precision studies were conducted in accordance with CLSI EP05 principles. These studies demonstrated excellent intra- and inter-assay reproducibility across both controlled spike-in experiments of H727 neuroendocrine tumor cells into stabilized whole blood and clinical samples. These cells are a well characterized, established well-differentiated neuroendocrine cell line originally derived from a human lung carcinoid [18]. In spike-in studies, coefficients of variation (CVs) for Ct values were <1% intra-assay and <2% inter-assay, while NETest2.0^®^ score variability remained low (2.1% and 4.1%, respectively). These results indicate minimal technical variability in amplification and scoring.

Importantly, clinical sample testing confirmed these findings under real-world conditions. No significant differences were observed between operators or across runs, and strong correlations were maintained (r = 0.89–0.94 for Ct values; r = 0.82–0.94 for scores). The stability of housekeeping gene expression further supports assay normalization robustness. Additionally, binary classification performance was perfect (100% concordance across 120 replicates), emphasizing the reliability of the assay in clinical decision-making contexts.

These findings align with CLSI EP05 expectations for precision and demonstrate that the assay maintains performance across sources of analytical variation, including operator, instrument, and time. Using Ct-based calculations consistent with CLSI EP17, the LoD was determined to be 2.2 cells/mL, and the LoQ 8.6 cells/mL. PROBIT modeling confirmed a 95% probability of detection at approximately 2.3 cells/mL, demonstrating concordance between statistical and Ct-based approaches. The distinction between LoD and LoQ reflects the expected increase in variance at the very low template concentrations inherent to qPCR assays. Above the LoQ, assay output demonstrated acceptable precision for quantitative reporting. These findings align with CLSI EP05 expectations for precision and demonstrate that the assay maintains performance across sources of analytical variation, including operator, instrument, and time. These also establish a defined analytical measurement capability and demonstrate reliable detection of low-abundance tumor-associated transcripts in peripheral blood. Detection of both housekeeping and target genes at concentrations as low as 1 cell/mL underscores the sensitivity of the assay platform. The concordance between Ct-based LoD and PROBIT-derived LoD further strengthens the robustness of the analytical model. Importantly, NETest2.0^®^ scores remained positive across the entire detection range, suggesting that the multigene algorithm integrates low-level signals effectively, mitigating stochastic variability that may affect single-marker assays. Although the assay output is ultimately expressed as an algorithm-derived score rather than a direct linear concentration, evaluation across serial cell dilutions demonstrated progressive increases in gene amplification and score output across the tested range, consistent with expected assay behavior. While a formal EP06 linear regression analysis is inherently limited for multigene algorithmic outputs, regression analysis across 1–100 cells/mL demonstrated a monotonic increase in score output (R^2^ = 0.91), without significant deviation from expected proportional response. These results are consistent with CLSI EP17 recommendations and support the assay’s suitability for early detection and monitoring applications where analyte levels may be low.

The stability of performance across days and operators is particularly relevant for clinical laboratories, where variability in reagent preparation, operator technique, and instrument cycling can influence RNA-based assays. The observed consistency indicates that the workflow—RNA extraction, reverse transcription, amplification, normalization, and algorithmic processing—is robust to routine operational variation. These findings support reproducibility suitable for longitudinal patient monitoring, where analytical drift could otherwise confound interpretation of serial measurements.

Analytical specificity and robustness were evaluated through interference testing using both endogenous and exogenous substances at clinically relevant concentrations. In accordance with CLSI EP07 principles for interference testing, common endogenous interferents (hemoglobin, bilirubin, triglycerides, urea, glucose) and commonly prescribed medications (aspirin, acetaminophen, ibuprofen, metformin, omeprazole) were assessed in spiked whole blood. First, demographic variables (age, sex, ethnicity) did not influence assay output, indicating robustness across patient populations. Second, endogenous and exogenous interference studies showed no significant impact on NETest2.0^®^ scores, with minimal bias (−3.94% and −1.65%, respectively).

Additionally, correlations between NETest2.0^®^ score output and standard hematologic parameters (red blood cells, white blood cells, neutrophils, lymphocytes, platelets) were evaluated in 130 NET patients. Importantly, no correlations were observed between NETest2.0^®^ scores and markers of renal or hepatic function, nor with blood cell counts. This suggests that physiological variation in circulating blood cell populations, e.g., the neutrophil-lymphocyte ratio [22] or numbers of circulating platelets [23] does not materially influence assay output. Similarly, no significant relationships were observed between assay output and renal or hepatic function markers. These findings collectively indicate that the mRNA assay, unlike other NET assays, e.g., Chromogranin A [24,25], is analytically stable across a broad range of physiological and biochemical conditions. The assay does not appear to be confounded by systemic physiological variation or hematologic composition, a common limitation in circulating biomarker assays. These data demonstrate resistance to common biochemical and pharmacologic confounders encountered in routine clinical specimens. These findings support compliance with CLSI EP07 principles and highlight the robustness of the multigene approach in minimizing susceptibility to biological and analytical interference.

Linearity and dynamic range were confirmed across a broad concentration range (0.01–200 ng/µL cDNA), with consistent gene amplification and stable NETest2.0^®^ scores between 1 and 200 ng/µL. While a plateau in scores was observed at higher cell concentrations (>100 cells/mL), this reflects expected biological saturation rather than analytical limitation. Reference gene stability per se, is critical in multigene expression assays [16,26]. The housekeeping genes demonstrated stable expression (CV% < 2.5%), high amplification efficiency, and minimal variability across tested conditions, supporting their suitability for normalization. Amplification efficiency across the 51 target transcripts was consistent, and nearly all targets were detectable in clinical samples. Inclusion of a standardized positive control on each PCR plate further strengthens internal quality control and mitigates batch-to-batch variability [27]. These features align with MIQE recommendations for qPCR assay design and documentation and support reproducibility of gene expression measurements. The ability to maintain consistent detection across several orders of magnitude demonstrates that the assay is well-suited for variable sample inputs, a critical requirement for clinical laboratory implementation. These findings align with CLSI EP06 guidance for linearity and confirm that assay performance is maintained within clinically relevant ranges.

Robustness was extensively evaluated under multiple conditions, including ambient storage, long-term freezing, and simulated transportation. RNA integrity remained stable over 10 days at room temperature and up to 5 years at −80 °C, with no significant changes in NETest2.0^®^ scores. These findings demonstrate exceptional sample stability, which is critical for decentralized collection and batch processing workflows. Transportation studies using ISTA 7D “Summer” and “Winter” profiles showed no degradation in RNA integrity or impact on assay performance. Classification accuracy remained 100% for both NET and control samples under these conditions, indicating that the assay is resilient to real-world logistical stressors. These results support compliance with CLSI EP25 (stability) and provide strong evidence for the assay’s suitability in routine clinical and reference laboratory settings.

The analytical sensitivity observed here is comparable to, and in some cases exceeds, that reported for other blood-based molecular assays targeting circulating tumor material, which typically demonstrate detection capabilities in the range of 1–10 tumor cells per milliliter [28]. Importantly, the assay achieves this sensitivity while maintaining low intra- and inter-assay variability. This balance between sensitivity and precision is essential for clinical deployment, as excessive analytical variability can negate gains in detection capability.

Several limitations should be acknowledged. First, although reproducibility was assessed across operators and instruments, all testing was conducted within a single laboratory. Formal multi-site reproducibility studies would further strengthen generalizability and are appropriate for future investigations. Second, spike-in experiments may not fully replicate the biological heterogeneity and fragmentation patterns of tumor-derived RNA in vivo. Third, the assay output is generated through a proprietary multigene algorithm. The algorithm remained locked during validation and demonstrated stable output across testing conditions, multivariate scoring systems inherently limit direct analytical decomposition of variance attributable to individual gene components. Forth, the assay uses the Nuclisafe™ Wren stabilization buffer tube. Studies are currently being undertaken to compare this tube with other commercially available RNA tubes for both stability as well as any impact on algorithmic output. The current metrics therefore relate to the validated NETest2.0^®^ collection and processing workflow.

NETest2.0^®^ utilizes standardized qPCR technology and centralized algorithmic analysis (see Figure 1). The assay workflow therefore is compatible with existing molecular laboratory infrastructure. Compared with repeated imaging studies or more complex sequencing-based approaches, the assay may offer a cost-effective strategy for longitudinal monitoring, although formal health-economic analyses remain warranted and are planned for future study.

## 5. Conclusions

The validation data demonstrated that NETest2.0^®^ met or exceeded the core analytical performance standards for molecular diagnostic assays. The assay exhibits defined analytical sensitivity (EP17), acceptable within- and between-run precision (EP05), stable response across a measurable range (EP06), and resistance to common endogenous and exogenous interferents (EP07). Performance was not materially influenced by demographic variables, routine laboratory parameters, operator handling, or instrument platform. Collectively, these characteristics establish the analytical robustness of the assay and provide the technical foundation required for standardized implementation in clinical molecular laboratories. The NETest2.0^®^ was shown as an analytically valid and reliable molecular diagnostic tool for neuroendocrine tumors.

## Figures and Tables

**Table 1:** Analytical validation focus and approaches.

No.	Validation	Focus	Approach	Measurement
1	Accuracy and Analytical Sensitivity	Evaluation of results (algorithm output) versus standard clinical reference methods (imaging and histological diagnosis) for NET disease detection and examination of specificity (results in non-NETs and in controls). Sub-analyses were performed on the NET cohort to examine whether type of NET, grade or metastases impacted diagnostic accuracy.	Clinical blood samples (NETs, non-NETs, controls), algorithm cut-off examination.	NETest2.0^®^ score
2	Precision (Repeatability and Reproducibility)	Intra-/inter-assay variability in the assay and testing across different operators, reagent lots, and time points for clinical samples.	NET cell line (extracted—positive control) and clinical samples. Two operators, 2 lots, 2 instruments.	Ct values NETest2.0^®^ score
3	Limit of Blank (LOB), Detection (LOD) and Quantification (LOQ)	The lowest amount of analyte that can be consistently detected and quantified.	Blood spike-in studies with NET cell line.	Ct values NETest2.0^®^ score
4	Analytical Specificity	The ability of the assay to unequivocally detect the target analyte in the presence of potentially interfering substances (e.g., proteins or genomic DNA from normal cells).	Blood spike-in studies with NET cell line (endogenous and exogenous interfering substances). Evaluation of clinical samples (correlation between scores and clinical parameters).	NETest2.0^®^ score
5	Linearity and Range	The ability of the assay to provide results that are directly proportional to the concentration of the analyte within a given range.	Blood spike-in studies with NET cell line.	Ct values NETest2.0^®^ score
6	Robustness and Stability	Demonstration of how long reagents and specimens remain stable under various storage and handling conditions.	Clinical blood samples—standardized transportation studies, ambient and −80 °C storage and freeze/thaw studies.	RIN values, Ct values, NETest2.0^®^ score

**Table 2:** Subjects evaluated for NETest2.0^®^ accuracy and sensitivity.

	NETs *^§^ (*n* = 568)	Other Cancers ** (*n* = 219)	Controls (*n* = 186)
Age (range)	62 (20–87)	64 (27–88)	55 (19–83)
Gender (M:F)	275:292	116:103	117:69
Ethnicity (A:B:H:W)	54:21:33:460	6:12:24:177	13:36:34:103

A = Asian, B = Black, H = Hispanic, W = White. * Includes: G1 (*n* = 253), G2 (*n* = 240), G3 (*n* = 41). Thirty-two had no grade data available. ^§^ Includes: Stage I–III (*n* = 236), Stage IV (*n* = 332). ** Other cancers included: lung—AC, lung—SCC (squamous cell carcinoma), esophagus, stomach, pancreas, appendix, colon, GIST (gastrointestinal stromal tumor), kidney, breast, prostate, melanoma.

**Table 3:** Clinical samples utilized for intra- and inter-analytical evaluation.

No.	Site	Age	Ethnicity	Gender	Sample ID	Original NETest2.0^®^ Score	Status
1	P	51	H	M	10003709B	<50	No Tx
2	SI	55	C	M	10003707B	≥50	SRD
3	P	73	C	M	10003744B	≥50	No Tx
4	SB	75	AA	M	10003747B	≥50	Stage IV, Tx
5	SB	81	A	M	10003778B	≥50	SRD
6	BP	64	C	F	10003824B	≥50	SRD, Tx

BP = bronchopulmonary (lung); P = pancreas; SI = small intestine. AA = African American; A = Asian; C = Caucasian; H = Hispanic. F = Female; M = Male. No Tx = not treated; SRD = stable residual disease; Tx = undergoing treatment.

**Table 4:** Sample characteristics of cases used to assess age, sex/gender and ethnicity.

Characteristic	Males (*n* = 393)	Females (*n* = 361)	*p*-Value
Median age (range) (years)	59 (19–87)	61 (21–87)	0.15 **
Ethnicity (A:B:H:W)	30:47:46:270	37:10:21:293	0.02 ^§^
Number treated (%) *	212 (53.9%)	169 (46.8%)	0.051 ^§^

* Treatment includes surgical (hemicolectomy, ablation, liver resection) and chemotherapeutic/biological (long-acting LAR) therapies. ** Calculated using 2-tailed Mann–Whitney test. ^§^ Calculated using Chi-square test (2-tailed). A = Asian, B = Black, H = Hispanic, W = White.

**Table 5:** Sample data (NETest2.0^®^ scores) by PCR QS7 instrument.

No.	Instrument	NETest2.0^®^ Score	*p*-Value *
1. 10003709B	1	8 ± 21	0.17
	2	0 ± 0	
2. 10003707B	1	78 ± 4.8	0.19
	2	76 ± 3.4	
3. 10003744B	1	76 ± 6.0	0.28
	2	79 ± 7.0	
4. 10003747B	1	78 ± 4.6	0.48
	2	77 ± 4.5	
5. 10003778B	1	75 ± 6.2	0.19
	2	71 ± 7.6	
6. 10003824B	1	80 ± 3.7	0.54
	2	79 ± 4.4	

* Mann–Whitney test.

## Data Availability

Due to privacy and ethical concerns, the data that supports the findings of this study are not publicly available but are available on request from the corresponding author.

## Abbreviations

The following abbreviations are used in this manuscript:

ALAT/ALT	alanine amino transferase
ALB	albumin
ALP	alkaline phosphatase
ASAT/AST	aspartate amino transferase
CLSI	Clinical and Laboratory Standards Institute
CV	co-efficient of variation
FOP	first operator
GFR	glomerular filtration rate
GGT	gamma-glutamyl transferase
GI	Gastrointestinal
GIST	Gastrointestinal stromal tumor
LDH	lactate dehydrogenase
LoB	limit of blank
LoD	limit of detection
LoQ	limit of quantification
mRNA	messenger RNA
miRNA	microRNA
NET	neuroendocrine tumor
qPCR	quantitative polymerase chain reaction
RBC	red blood cell
RCC	Renal clear cell cancer
SOP	second operator
WBC	white blood cell
